# Antecedent cancer in Takotsubo syndrome predicts both cardiovascular and long-term mortality

**DOI:** 10.1186/s40959-019-0053-6

**Published:** 2019-11-22

**Authors:** Thanh H. Nguyen, Jeanette Stansborough, Gao J. Ong, Sven Surikow, Timothy J. Price, John D. Horowitz

**Affiliations:** 1 Cardiology Unit, Department of Cardiology, The Queen Elizabeth Hospital, 28 Woodville Road, Woodville South, SA 5011 Australia; 2 The University of Adelaide, Adelaide, Australia; 3 Department of Medical Oncology, Queen Elizabeth Hospital, Woodville South, Australia

**Keywords:** Takotsubo syndrome, Antecedent cancer, Cardiovascular mortality

## Abstract

**Background:**

Takotsubo syndrome (TTS), primarily an acute myocardial inflammatory condition engendered by catecholamine exposure, is associated with similar long-term mortality rates to those of patients with acute myocardial infarction. However, there is increasing evidence of a nexus between TTS and underlying malignancies:- many patients have antecedent cancer (A/Ca), while incremental risk of late cancer-related death has also been reported.

**Purpose:**

To evaluate potential interactions between A/Ca among TTS patients and both early and late clinical course.

**Methods:**

Three hundred forty-six consecutive TTS patients [aged 69 ± 13 (SD) years, males: 8.2%] were prospectively followed up for a median duration of 4.1 (IQR 2.2–6.4) years. Associations between A/Ca and severity of acute attacks, in-hospital complications and long-term death rates were sought utilising univariate analyses followed by multiple logistic regression analysis.

**Results:**

A/Ca (present in 16.8% of patients) was associated with (i) greater elevation of hs-CRP and NT-proBNP concentrations (*p* = 0.01 and 0.04, respectively), (ii) more complicated in-hospital clinical course, with major adverse cardiac events (MACE) in 30.9% of patients, compared to 18.2% in non-A/Ca patients (*p* = 0.04). Long-term all-cause mortality rate was also greater [hazard ratio (HR) = 2.4, *p* = 0.0001] in A/Ca patients, with an excess cardiovascular (CVS) fatality rate (HR = 3.1, *p* = 0.001). On multivariate analysis, male gender, peak plasma concentrations of normetanephrine and hs-CRP, early arrhythmias and development of shock, but not A/Ca per se*,* were all independently associated with increased long-term mortality rate. Furthermore, patients discharged on β-adrenoceptor antagonists (βBl) or angiotensin converting enzyme inhibitors/ angiotensin receptor blockers (ACEi/ARB) had lower long-term mortality rates (β = − 0.2, *p* = 0.01; β = − 0.14, *p* = 0.05, respectively).

**Conclusions:**

(1) A/Ca is associated with greater clinical severity of initial TTS attacks and substantially greater long-term CVS-related as well as all-cause mortality.

(2) Post-discharge therapy with either βBl or ACEi/ARB is associated with reductions in long-term mortality rates.

Overall, the current data suggest operation of substantial interactions between neoplasia and TTS, both at the level of pathogenesis and of outcomes.

## Introduction

Takotsubo syndrome (TTS) occurs predominantly in aging women and largely reflects acute catecholamine-induced myocardial inflammation [1]. Only approximately 4% of TTS patients die in hospital at initial presentation, but long term mortality rates are similar to those for acute myocardial infarction patients [2, 3].

The precise range of stimuli for development of attacks of TTS remains uncertain, as does the basis for predominance of female gender and advanced patient age. There is increasing evidence that inflammation of the myocardium may be triggered by Gi-biased post-receptor responses to β_2_-adrenoceptor agonist stimulation in susceptible individuals [4, 5], and that excessive β_2_-adrenoceptor [6, 7] triggered nitric oxide release may initiate nitrosative stress and thus inflammation. However, precisely why this tends to occur in aging women remains uncertain.

Recently, reports have been made from several groups, including the International InterTAK collaboration, that TTS is often associated with presence of internal malignancy at the time of presentation [8–10]. Furthermore, there is a suggestion that TTS represents a basis for increased risk of later emergence of malignancies and for fatal outcomes from those malignancies [11, 12]. Specifically, it has been suggested that a higher proportion of patients die of cancer following TTS than following acute myocardial infarction [13, 14]. Registry data [10] suggest that approximately 39% of deaths following TTS are attributed to cancer. These findings therefore suggest some commonality of pathogenesis between TTS and internal malignancies, and it has even been suggested that TTS, should be regarded as a paraneoplastic disease state [14].

If the presence of cancer were a predisposing factor for initial attacks of TTS, it might also represent a basis for increased severity of these index episodes. Furthermore, it could be argued that the continued presence of cancer might affect long-term patient outcomes, both directly and via an interaction with cardiovascular (CVS) homeostasis.

In the current study, we therefore sought to evaluate the impact of antecedent cancer (A/Ca) on short and long-term outcomes in a registry-based cohort of TTS patients who were prospectively followed up from the time of diagnosis.

The principal objective of the study was to determine the impact of A/Ca on long-term all-cause, CVS, and non-CVS mortality rates.

The results suggest that A/Ca exerts reciprocal and ongoing deleterious interactions with TTS, and that these interactions may be important in the management of such patients. Furthermore, the results point to a substantial impact of some forms of pharmacological management on long-term mortality rates following TTS.

## Methods

### Patient population

Screening for TTS was performed routinely on patients admitted to 3 tertiary referral hospitals in Adelaide, South Australia. The diagnosis of TTS was based upon modified Mayo Clinic Criteria [15], essentially consisting of presence of evidence of acute left ventricle (LV) regional systolic dysfunction, together with exclusion of acute myocardial infarction (AMI) by coronary angiography and/or cardiovascular magnetic resonance (CMR) [16]. The severity of acute attacks was quantitated via: (1) severity of LV dysfunction [acute LV ejection fraction (LVEF), as measured by transthoracic echocardiography], (2) clinical measures of hemodynamic changes (lowest systolic BP within the first 48 h, presence of shock), (3) extent of myocardial injury (peak troponin T concentrations), (4) inflammatory changes [peak concentrations of high sensitive C-reactive protein (hs-CRP) and N-terminal pro B-type natriuretic peptide (NT-proBNP)], and (5) markers of catecholamine release (normetanephrine and metanephrine concentrations). Patients in whom TTS emerged in the context of an acute life-threatening extracardiac disease and/or medical procedure were classified as “secondary” TTS [17].

At the time of discharge following initial admission with TTS, demographic data, including the prior diagnosis of A/Ca, patients’ medications and, where appropriate, anti-cancer treatment, were recorded.

This investigation was approved by the Institutional Ethics of Human Research Committee (Central Northern Adelaide Health Service: the Queen Elizabeth Hospital and Lyell McEwin Hospital; protocol number: 009014). Written informed consent was obtained from all participants before study entry.

### Patients’ follow-up

Patients were routinely seen at 3 months, and annually thereafter. Data regarding patient outcomes were obtained both by direct follow-up and by recourse to national mortality data. Primary causes of death were categorised as documented on death certificates, while late emergence of malignancies was documented on the basis of follow-up. Recurrences of TTS were documented from both hospital admission data and via follow-up.

### Major adverse cardiac events (MACE)

The objectives of the study were to determine whether A/Ca had any impact on:-

- In-hospital course: In-hospital MACE were classified as the combination of shock, arrhythmias and death.
- Long-term mortality rate (including both all-cause and CVS mortality rates)

### Statistics

Data were analyzed using SPSS software (version 23, Chicago, Illinois, USA) and presented as mean and SD or median and interquartile range depending on data distributions.

Comparisons between TTS patients with and without A/Ca regarding markers of severity of acute attacks, were performed utilizing non-paired t-tests or Wilcoxon rank sum tests as appropriate. Comparisons of proportional data were analyzed utilizing Chi-squared or Fisher’s exact tests as appropriate.

Impact of A/Ca on mortality rates was evaluated via Kaplan-Meier analyses and a univariate Cox proportional hazards model.

Associations between markers of severity of index attacks and long-term mortality rates were sought utilising univariate analysis, followed by backwards stepwise multiple logistic regression analysis. Variables prospectively selected for these analyses (in accordance with hypotheses to be tested) were patients’ age, sex, presence of A/Ca, CVS risk factors, acute plasma concentrations of NT-proBNP, hs-CRP, normetanephrine, troponin-T, development of shock, arrhythmias, primary versus secondary TTS, TTS recurrence, and discharge medications [β-adrenoceptor antagonists (βBl) and angiotensin converting enzyme inhibitors/ angiotensin receptor blockers (ACEi/ARB)].

A *p* value of < 0.05 was considered significant.

## Results

### Patients’ characteristics

The clinical characteristics of the study population are described in Table 1.

**Table 1:** TTS patients’ characteristics: Entire cohort and subdivision according to previous diagnosis of A/Ca. Statistical comparisons are between A/Ca and no A/Ca subgroups

	Entire group (*n* = 346)	A/Ca (*n* = 58)	No A/Ca (*n* = 288)	*p*
Duration of follow-up (years)	4.1 (2.2–6.4)	3.5 (1.2–5.8)	4.4 (2.3–6.8)	0.05
Age (years)	69 ± 13	74 ± 10	68 ± 14	0.001
Male: (%)	8.2	14.3	7.0	NS
Secondary TTS (%)	34	47	31	0.02
Site (Apex; %)	66.4	64.3	66.7	NS
Annual recurrence rate (%)	2.1	1.8	2.2	NS
CVS risk factors				
Hypertension (%)	56.0	54.7	57.2	NS
Diabetes mellitus (%)	18.2	17	18.4	NS
Dyslipidemia (%)	37.8	34.0	38.6	NS
Current smoking (%)	9.8	9.4	9.9	NS
Markers: size of acute attacks				
Minimal systolic BP (mmHg)	97 ± 16	98 ± 15	96 ± 16	NS
Acute LVEF (%)	46 ± 12	44 ± 13	47 ± 12	NS
Peak NT-proBNP (ng/L)	4800 (2600–9000)	5800 (3600–10,800)	4600 (2500–8600)	0.04
Peak normetanephrine (pmol/L)	980 (610–1400)	960 (600–1460)	980 (620–1410)	NS
Peak metanephrine (pmol/L)	200 (200–270)	210 (200–290)	200 (200–270)	NS
Peak hs-CRP (mg/L)	13 (6–44)	23 (8–100)	11 (6–38)	0.01
Peak troponin T (ng/L)	400 (223–620)	310 (187–564)	400 (230–638)	NS
In-hospital complications (%)				
Arrhythmias	15.1	21.8	13.8	NS
Shock	7.4	12.7	6.4	NS
Mortality	3.2	8.9	2.1	0.02
MACE	20.3	30.9	18.2	0.04
Neoplasia (%)				
A/Ca (Prior/current)	16.8			
Chemo/Immunotherapy		34.6		
Subsequent neo malignancy		6.9		
Discharge CVS medications (%)				
ACEi/ARB	78.4	88.2	76.5	0.07
βBl	42.6	31.3	44.7	0.09
Statins	50.5	45.1	51.5	NS
Aspirin	42.4	37.3	43.4	NS

Of 346 TTS patients, there were 58 with A/Ca (16.8% of the total cohort). Patients with A/Ca were significantly older that those without A/Ca [74 ± 10 vs. 68 ± 14 (SD) years, *p* = 0.001], but did not differ significantly from other TTS patients as regards site within the LV of hypokinesis, CVS risk factors and mean initial systolic blood pressure. A larger proportion of A/Ca patients had secondary TTS, and plasma concentrations of NT-proBNP and hs-CRP were significantly greater in A/Ca patients (*p* = 0.04 and 0.01, respectively).

The majority of patients were discharged on cardio-active medications. Approximately 78% received either ACEi or ARB, while 43% received βBl. Statins and aspirin were also extensively prescribed.

Approximately 35% of patients with A/Ca had received treatment with either chemotherapeutic or immunotherapeutic drugs (Table 1). In general, a substantial period of time had elapsed since the initial diagnosis of cancer:only two patients were receiving chemo/immunotherapy at the time of TTS admission. The most common internal malignancy was breast cancer, accounting for 33% of total A/Ca (Table 2). Only 3 patients had received anthracyclines and 2 trastuzumab as components of chemotherapy.

**Table 2:** Distribution of A/Ca and subsequent de novo malignancies within the studied cohort

Type of cancer	A/Ca	Subsequent cancer^a^
Breast	19	1
Uterine/Cervical/endometrial	5	2
Ovarian	0	2
Prostate	5	0
Lung	7	7
Skin	7	2
Bowel	9	5
Gastric carcinoid	1	0
Esophageal	0	1
Liver	0	1
Pancreatic	0	2
Metastatic SCC tongue	1	0
Thyroid	0	1
Parotid tumor	1	0
Phaeochromocytoma	2	1
Bladder	0	1
CLL	1	1

^a^Subsequent cancer are de novo malignancies

### Early and late clinical course

#### In-hospital MACE

Overall in-hospital mortality was 3.2% (Table 1) which was similar to that described in previous larger studies [2, 17]. There was a significant increase in in-hospital mortality rate in patients with A/Ca (*p* = 0.02). Early MACE occurred in 30.9% of patients with A/Ca, compared to 18.2% in patients with no A/Ca (*p* = 0.04) (Table 1). Early arrhythmias particularly tachyarrhythmias, occurred commonly: (new AF/flutter [*n* = 23], supraventricular tachycardia [*n* = 9], ventricular tachycardia/ fibrillation [*n* = 12]) with a non-significant excess in the A/Ca group.

#### Subsequent course

Median duration of follow-up was 4.1 (IQR 2.2–6.4) years and was shorter for A/Ca patients (*p* = 0.05), because of greater mortality rates. During follow-up, a total of 27 (7.8%) patients were diagnosed with “new” internal malignancies (Table 2). In a further 11 cases, clinical emergence of metastasis occurred among patients with A/Ca.

A total of 59 patients died during the course of the study, with 15 (25%) of deaths attributed to cancer and 30 (51%) to CVS disease states.

#### Impact of A/Ca on subsequent clinical course

This analysis was conducted to determine whether A/Ca might in any way predispose patients either to recurrence of TTS or to CVS death. Comparisons were made between subgroups with and without A/Ca. Hazard ratio (HR) for relative risks were derived from total subgroup data sets.

##### Recurrence

Recurrence of TTS occurred in 8.5% of the total cohort and 7.3% of patients with A/Ca (annual recurrence rates 1.9 and 2.1%, respectively, *p* = NS). No recurrences were associated with in-hospital death.

##### Mortality

**Fig. 1:**
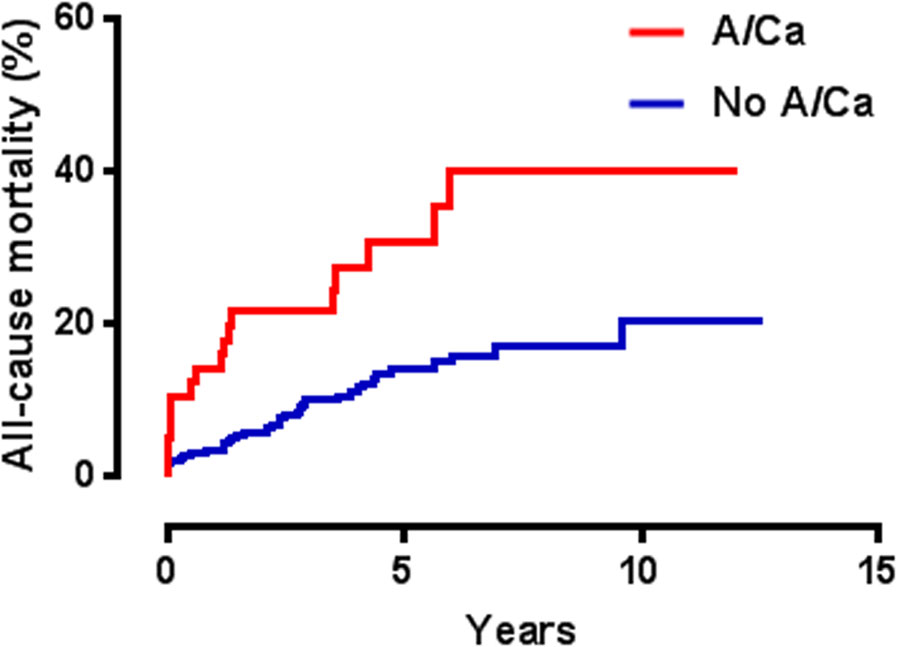
Kaplan-Meier analysis indicating increased of long-term all-cause mortality in patients with A/Ca [hazard ratio (HR) = 2.4, *p* = 0.0001]

**Fig. 2:**
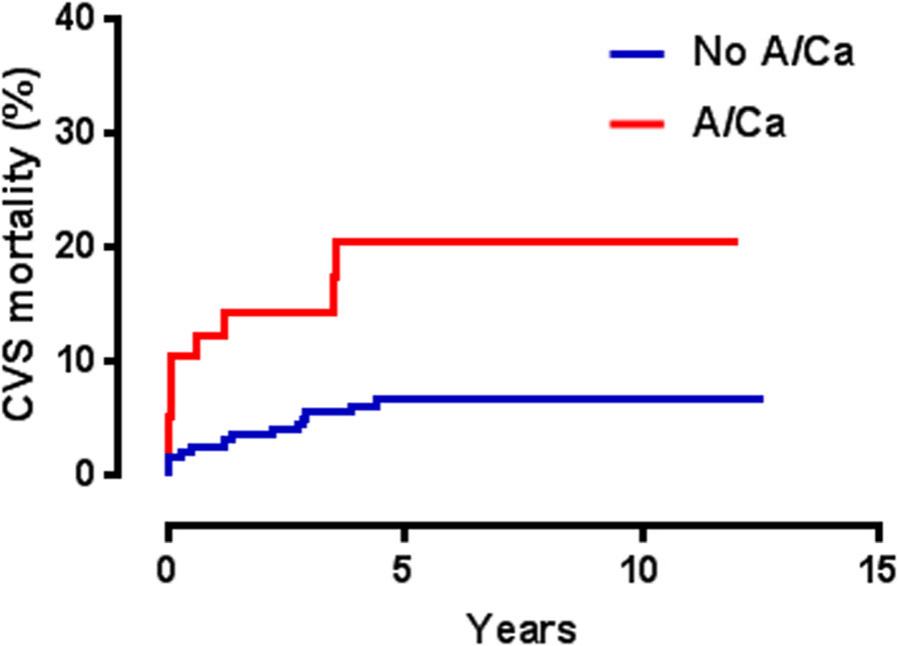
Kaplan-Meier analysis indicating greater CVS mortality in patients with A/Ca [hazard ratio (HR) = 3.1, *p* = 0.001]

Both long-term all-cause mortality (HR = 2.4, *p* = 0.0001) (Fig. 1) and CVS death rates were substantially greater (HR = 3.1, *p* = 0.001) in patients with A/Ca (Fig. 2).

### Factors predictive of all-cause mortality

**Fig. 3:**
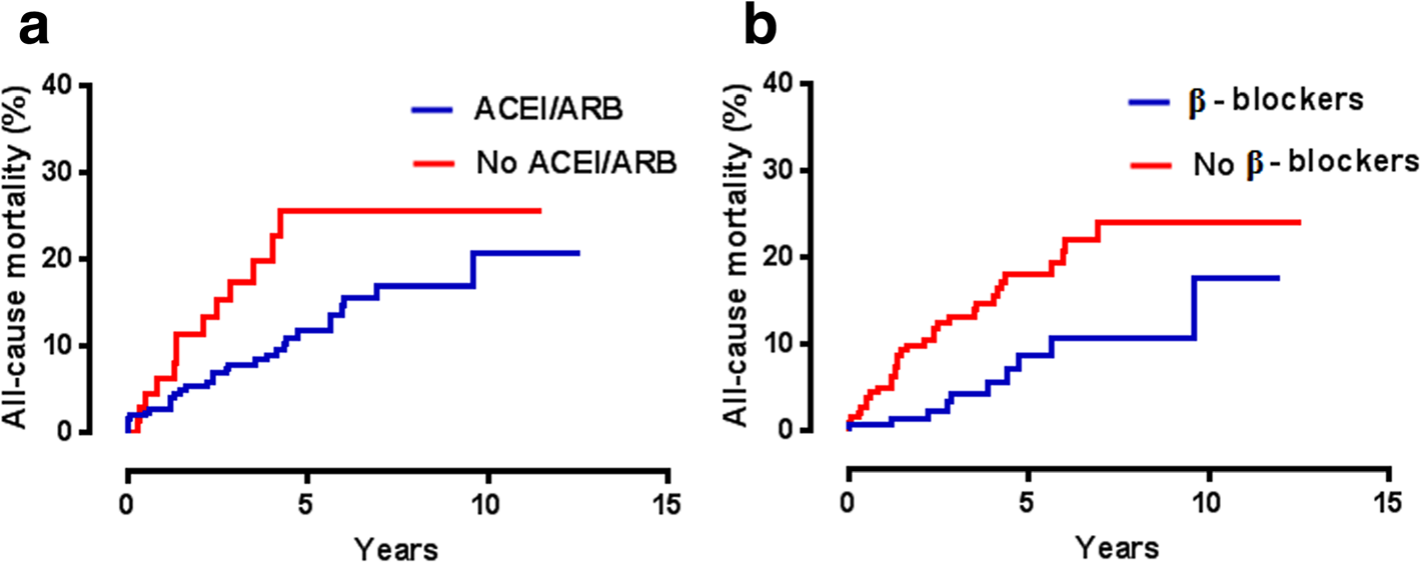
Kaplan-Meier analyses: associations between discharge treatment with ACEi/ARB (**a**; *p* = 0.03) and βBl (**b**: *p* = 0.01) and long-term all-cause mortality rates

As shown in Fig. 3a and b, discharge prescription of ACEI/ARB or of βBl was associated with lower mortality rates (*p* = 0.03 and 0.01, respectively) on univariate comparisons. Interestingly, the relative impact of these forms of therapy on mortality among A/Ca patients, evaluated by Fisher’s exact test, was significantly greater for patients prescribed with ACEI/ARB (*p* = 0.02).

Furthermore, backwards stepwise multiple logistic regression analysis (Table 3) revealed that extent of elevation of plasma levels of normetanephrine, presence of early arrhythmias, early development of shock, male gender, as well as ACEi/ARB or βBl therapies at discharge but not A/Ca per se, were independently associated with increased long-term mortality rates.

**Table 3:** Predictors of long-term all-cause mortality in the entire TTS patient cohort: results of backwards stepwise multiple logistic regression analysis

Predictors	β	*p*
Male gender	0.15	0.04
Presence of shock during acute admission	0.15	0.04
Peak normetanephrine concentrations	0.25	0.001
Peak hs-CRP concentrations	0.13	0.09
Arrhythmias at admission	0.21	0.006
βBl at discharge	−0.2	0.01
ACEi/ARB at discharge	−0.14	0.05

*ACEi/ARB* Angiotensin converting enzyme inhibitors/ Angiotensin receptor blockers

## Discussion

**Fig. 4:**
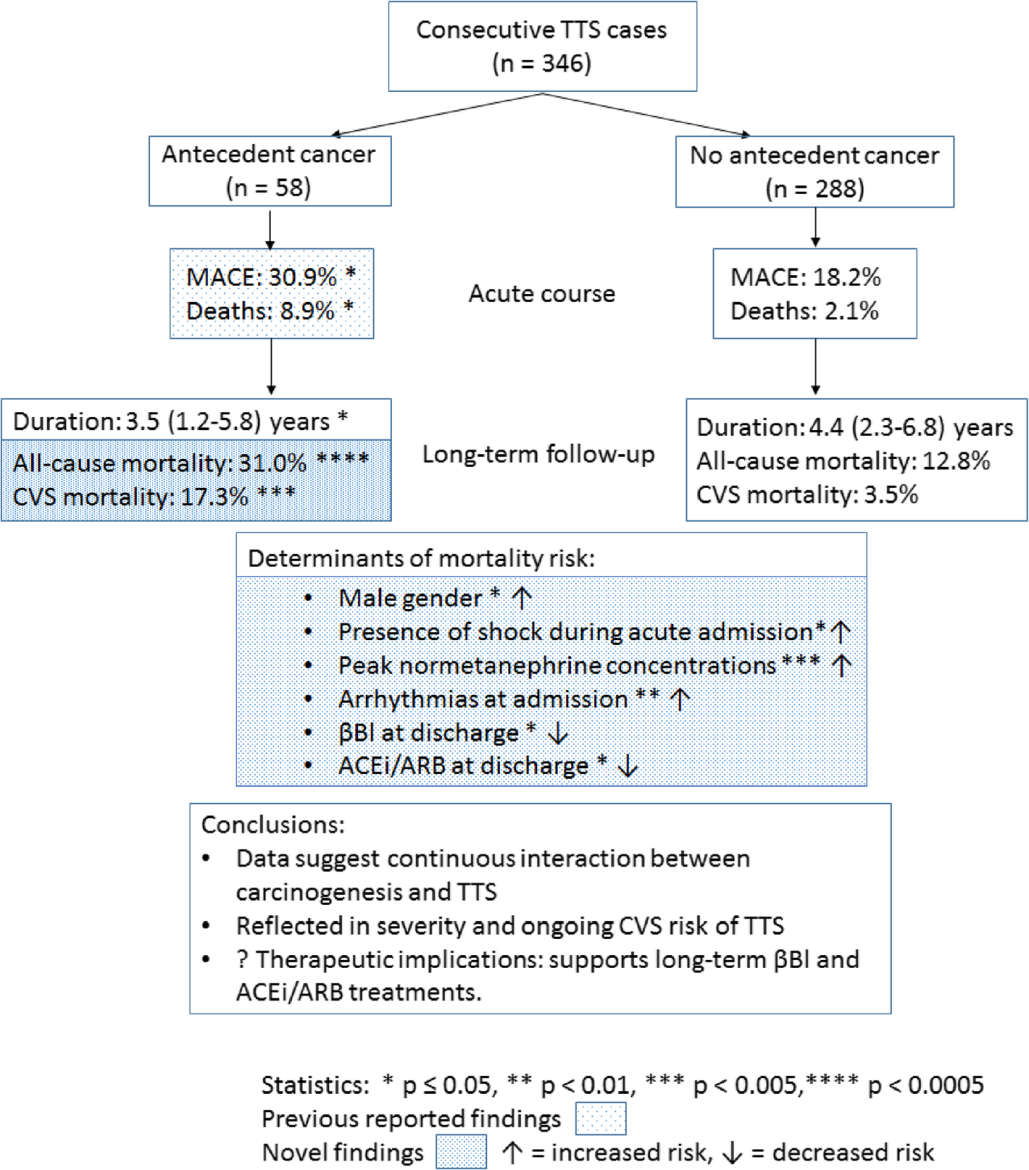
Schematic of study design and major findings

The structure and main findings of the current investigation are summarised in Fig 4.

The results of this study are important because:

- They confirm that a substantial proportion of patients with TTS have known A/Ca, and demonstrate that breast cancer is the most common association.
- They show that TTS in association with A/Ca more often presents as secondary TTS,with associate clinical impact includingsignificantly increased in-hospital MACE rates [17]. Indeed, Cammann et al. [8] have recently reported, within the InterTAK cohort, increased in-hospital death rates in patients with A/Ca and TTS.
- They also show that patients with A/Ca have greater risks not only of late all-cause mortality, but somewhat surprisingly, a markedly increased risk of CVS death.
- On multivariate analyses, factors predicting long-term mortality include male gender, extent of catecholamine release (normetanephrine concentrations), acute attack hemodynamic impact (presence of shock, early arrhythmias),and extent of inflammatory activation (hs-CRP concentrations).
- Patients discharged on ACEi/ARB or on βBl had substantially lower mortality rates, and this apparent influence of discharge medication on survival was most marked among A/Ca patients who were prescribed ACEi/ARB.

Thus, findings (2) and (3) point strongly to some substantial and ongoing interaction between the presence of cancer and the probability of CVS complications (short- and long-term) of TTS. To the best of our knowledge, this is the first time that such an association has been reported.

The results also suggest that there are reciprocal long-term interactions between CVS outcomes and presence of A/Ca, in the sense that CVS death rates were substantially elevated in patients with A/Ca. Previously, it has been observed that patients with TTS have an increased risk of long-term cancer death relative to control populations. This was not strongly suggested by the current data, but no control population was used. The data regarding excess long-term CVS mortality in A/Ca patients, were statistically robust, but no complete explanation for the finding is currently available. One possible explanation would be related to patients’ age (older for A/Ca patients) and/or comorbidities. However, patients with A/Ca had similar CVS risk profiles (with the exception of age) to those without A/Ca, and patients’ age was not an independently significant predictor of mortality.

**Fig. 5:**
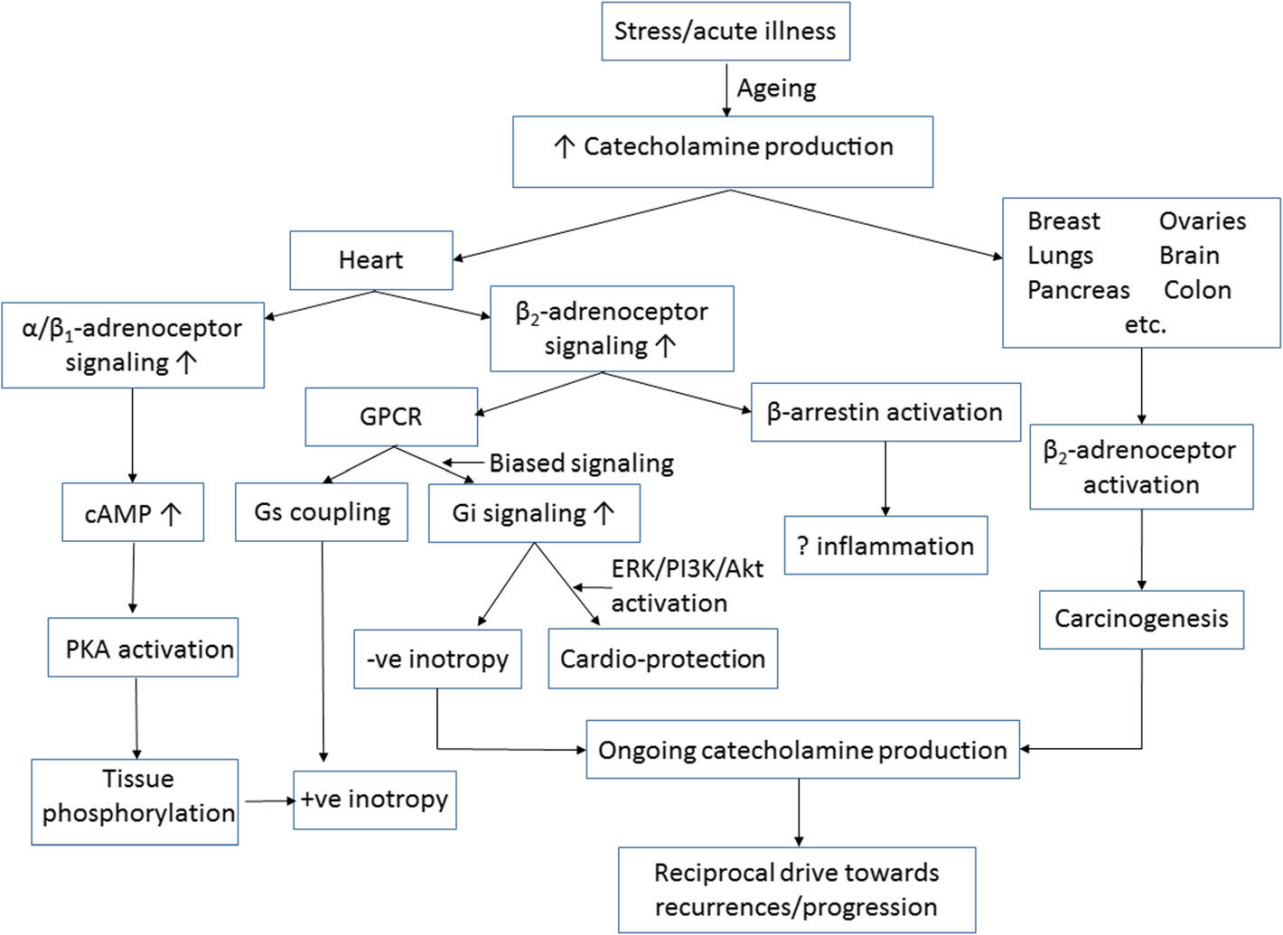
Schematic: potential signal-transduction pathways contributing to (i) shared pathogenesis between TTS and systemic malignancies and (ii) ongoing interactions between presence of malignancy and cardiovascular outcomes post TTS

It could be also be argued that the “main” finding might have related to greater hemodynamic impact of the acute attack in patients with A/Ca, leading putatively to greater long-term myocardial fibrosis [18] and therefore greater risk of late cardiac failure and death. Indeed, the available data (see Table 1) suggest that hemodynamic impact might have been greater in A/Ca patients, but this was not studied in detail. A recent analysis from the InterTAK group [19] also demonstrated that clinical factors associated with haemodynamic impact of TTS attacks, including hypotension, tachycardia and reduced left ventricular ejection fraction, all function as adverse long-term prognostic markers. Other recent publications [20, 21] also documented that patients with A/Ca had poor in-hospital outcomes. As a number of neoplasms may be associated with increased catecholamine production, the associated neoplasms themselves may have contributed to severity of attacks, as schematized in Fig. 5. What is unlikely is that our observations were primarily related to the impact of heart failure induced by prior chemotherapy, as only a minority of patients had received chemotherapy and only 2 were receiving concurrent chemotherapy. Therefore, although precipitation of TTS is a well-recorded complication of cancer chemotherapy, direct contributions of chemotherapy per se to emergence of TTS are likely to be relevant only to a small proportion of TTS patients.

Discharge prescription of either ACEi/ARB or βBl predicted low mortality risk both on univariate (Fig. 3) and on multivariate analysis (Table 3). There are theoretical reasons why ACEi/ARB treatment might reduce risk of inflammation and therefore impact of TTS [22]. Furthermore, a previous meta-analysis [23] suggested that ACEi/ARB therapy reduces risk of TTS recurrence; in the current study, the observed annual rates of recurrence were 2.2 and 1.8% for patients with and without A/Ca, respectively (*p* = NS). The salutary effects of βBl therapy (Fig. 3b, Table 3) were a little surprising, given the relative β_1_-adrenoceptor selectivity of most currently used βBl and the implication from animal studies that the signal transduction pathway for catecholamine-induced precipitation of TTS is primarily or exclusively related to β_2_-adrenoceptor stimulation [4, 5]. Previous clinical studies [3, 10, 24] have failed to document any benefit associated with discharge therapy including βBl, but this issue now merits re-examination. Nevertheless, in a non-randomized data set, it remains possible that discharge prescription of βBl was biased towards “less ill” patients. Interestingly, the prognostic benefit of ACEi/ARB therapy seemed to be most marked in A/Ca patients.

The study, while representing the first of its type has a number of additional limitations.

It is possible that there may have been some inaccuracies in attribution of cause of death, and also that patient pharmacotherapies at discharge might not have been properly representative of long-term treatment.

No firm conclusions can be drawn about impact on recurrence rates, given the low number of diagnosed recurrences, the possibility that some recurrences were fatal, and the extensive use of ACEi/ARB, which may have reduced likelihood of recurrence [23].

On backwards stepwise multiple logistic regression, A/Ca per se was not a significant correlate of mortality risk. The most likely theoretical explanation for this finding was that A/Ca was an indirect factor predisposing to mortality, partially via increased severity of acute TTS episodes, as evidenced by the identification of acutely elevated plasma levels of normetanephrine and hs-CRP, presence of early arrhythmias and shock, and male gender as significant multivariate correlates of mortality. Plasma normetanephrine concentrations probably relate to severity of attacks both via intensity of initial catecholamine discharge and via its autonomic consequences of persistent sympathetic activation (for example in the face of hypotension). In the event, however, patients with A/Ca differed primarily on the basis of more intense early inflammation, as characterized via hs-CRP and NT-proBNP elevation (Table 1). Furthermore, there is a possibility that measurement of other parameters, such as liver/renal function tests and white blood cell count, may have shed additional insight on determinants of prognosis.

As regards mechanisms of association with CVS mortality risk, relevant data remain very limited, and no particular type of cancer predominated. While chronic inflammation may represent a risk factor for both carcinogenesis and development of ischemic heart disease [25], the current data add little beyond that.

How, then, might an association between A/Ca and long-term CVS mortality risk have operated? One possibility is that both index and recurrent attacks are more likely to occur in patients with cancer. In the current series, there were too few recurrent episodes to be certain about this possibility. Furthermore, it remains possible that some of the late CVS deaths might have reflected arrhythmias associated with recurrence.

A second possibility would be the induction of TTS as a complication of antineoplastic therapy [26] or because of prior cardiotoxicity induced by antineoplastic drugs. If this contributed to the current findings, it would probably have been a relatively minor factor, given that only 34.5% of patients with A/Ca had received chemo/immuno therapies.

A final possibility is of a “hormone-like” effect of some cancers inducing cardiotoxicity. There is evidence that many cancers may exhibit paracrine effects [27]. In general, such effects are potentially independent of the induction of TTS, or of the mechanisms, including Gi-protein biased post-receptor β_2_-adrenoceptor signalling and induction of nitrosative stress [7, 28], thought to underlie the pathogenesis of TTS. Figure 5, a schematic view of potential reciprocity between TTS and cancer both at the level of initiation and progression, represents an integrated view of our current mechanistic speculation in this regard.

However, it is also known that both ovarian and breast cancers may rely crucially on induction of activity of poly (ADPribose)polymerase-1 (PARP-1) [29], which appears to play a crucial role in myocardial energy impairment in a rat model of TTS [7]. To date, there is no suggested mechanism to explain multi-organ induction of PARP-1 activity, so this possibility remains to be tested.

In conclusion, A/Ca is a common finding among patients with TTS, and is associated with a more complicated in-hospital course, and a far higher long-term mortality risk, towards which ongoing cardiac events contribute substantially. While the mechanisms underlying this evidence of close ongoing association and probable reciprocity between TTS and cancer must remain somewhat speculative at this stage, at the very least the data would suggest that A/Ca be viewed as a high risk indexation factor among TTS patients.

The further finding that treatment with ACEi/ARB or βBl appears to be protective in long-term follow-up of TTS patients cannot be regarded as a substitute for randomized clinical trials, and indeed no such trials have yet been completed. However, coupled with the results of the previous meta-analysis [17], these data add to the argument for such a study to be undertaken.

## Data Availability

The datasets used and/or analysed during the current study are available from the corresponding author on reasonable request.

## Abbreviations

A/Ca: Antecedent cancer
ACEi: Angiotensin converting enzyme inhibitor
ARB: Angiotensin receptor blocker
BP: Blood pressure
CVS: Cardiovascular system
hs-CRP: high sensitive C-reactive protein
LVEF: Left ventricular ejection fraction
MACE: Major adverse cardiac events
NT-proBNP: N-terminal-pro-B-type natriuretic peptide
TTS: Takotsubo syndrome
βBl: β-adrenoceptor antagonist
